# Can Incorporating Molecular Testing Improve the Accuracy of Newborn Screening for Congenital Adrenal Hyperplasia?

**DOI:** 10.1210/clinem/dgae297

**Published:** 2025-03-17

**Authors:** Kyriakie Sarafoglou, Amy Gaviglio, Carrie Wolf, Cindy P. Lorentz, Aida Lteif, Jennifer Kyllo, Gretchen Radloff, Zachary Detwiler, Carla D. Cuthbert, James S. Hodges, Scott D. Grosse, Christopher N. Greene, Suzanne Cordovado

**Affiliations:** 1 Department of Pediatrics, University of Minnesota Medical School, Minneapolis, MN 55454, USA; 2 Department of Experimental and Clinical Pharmacology, University of Minnesota College of Pharmacy, Minneapolis, MN 55455, USA; 3 Newborn Screening Program, Minnesota Department of Health, St. Paul, MN 55155, USA; 4 4ES Corporation, San Antonio, TX 78261, USA; 5 Department of Pediatrics, Mayo Clinic College of Medicine, Rochester, MN 55905, USA; 6 Department of Endocrinology, Children’s Hospitals and Clinics of Minnesota, St. Paul, MN 55102, USA; 7 Centers for Disease Control and Prevention, Atlanta, GA 30333, USA; 8 CRISPR Therapeutics Inc, Boston, MA 02127, USA; 9 School of Public Health, University of Minnesota Division of Biostatistics, Minneapolis, MN 55455, USA

**Keywords:** congenital adrenal hyperplasia, newborn screening, premature infants, *CYP21A2* gene

## Abstract

**Context:**

Single-tier newborn screening (NBS) for congenital adrenal hyperplasia (CAH) using 17-hydroxyprogesterone (17OHP) measured by fluoroimmunoassay (FIA) in samples collected at 24 to 48 hours produces a high false-positive rate (FPR). Second-tier steroid testing can reduce the FPR and has been widely implemented.

**Objective:**

We investigated the accuracy of an alternative multitier CAH NBS protocol that incorporates molecular testing of the *CYP21A2* gene and reduces the first-tier 17OHP cutoff to minimize missed cases.

**Methods:**

We create a Minnesota-specific *CYP21A2* pathogenic variants panel; developed a rapid, high-throughput multiplex, allele-specific-primer-extension assay; and performed a 1-year retrospective analysis of Minnesota NBS results comparing metrics between a conventional steroid-based 2-tier protocol and a molecular-based multitier NBS protocol, applied post hoc.

**Results:**

*CYP21A2* gene sequencing of 103 Minnesota families resulted in a Minnesota-specific panel of 21 pathogenic variants. The Centers for Disease Control and Prevention created a molecular assay with 100% accuracy and reproducibility. Two-tier steroid-based screening of 68 659 live births during 2015 resulted in 2 false negatives (FNs), 91 FPs, and 1 true positive (TP). A 3-tier protocol with a lower first-tier steroid cutoff, second-tier 21-variant *CYP21A2* panel, and third-tier *CYP21A2* sequencing would have resulted in 0 FNs, 52 FPs, and 3 TPs.

**Conclusion:**

Incorporation of molecular testing could improve the accuracy of CAH NBS, although some distinct challenges of molecular testing may need to be considered before implementation by NBS programs.

Congenital adrenal hyperplasia (CAH) due to 21-hydroxylase deficiency (21OHD) is a form of primary adrenal insufficiency characterized by impaired cortisol production leading to increased 17-hydroxyprogesterone (17OHP) and adrenal androgen synthesis. CAH is caused by pathogenic variants in the *CYP21A2* gene and is classified in order of severity of affected enzyme activity from the 2 classic (severe) forms, salt-wasting (SW) and simple-virilizing (SV), to the milder non-classic (NC) form. CAH can manifest in the newborn period with life-threatening SW and/or adrenal crises, varying degrees of female genital virilization, and/or incorrect sex assignment. The prevalence of classic CAH based on 3 years of US newborn screening (NBS) data was 1 in 14 300 (1, 2).

NBS for classic CAH is performed by all US states and in many other countries. The primary NBS method for identifying CAH is a single 17OHP measure by fluoroimmunoassay (FIA) from a dried blood spot (DBS), which in most US states is typically collected on filter paper between 24 and 48 hours of life. This assay has a high rate of false positives (FPs), particularly in premature infants (3–9). Without retesting low birth weight infants, the positive predictive value (PPV) may be less than 2%, and even with retesting is less than 10% (10). To reduce FPs from 17OHP testing, several states have introduced second-tier screening using liquid chromatography–tandem mass spectrometry (LC-MS/MS) for steroid profiling with varying degrees of improvement (11, 12). It is commonly assumed that false-negative (FN) NBS results for classic CAH are rare and that sensitivity is close to 100%. However, several studies that have conducted systematic surveillance have reported FN rates with one-tier 17OHP testing at 1 to 2 days of life ranging from 2.5% to 30% (4, 6, 7). Several states that routinely collect and test repeat specimens at approximately age 2 weeks have reported as many as one-third of cases of CAH missed on the first screen (13, 14). In Minnesota, even following the introduction of second-tier steroid testing using sensitive assays such as a diethyl ether extraction assay or LC-MS/MS to measure 17OHP, androstenedione (D4A), and cortisol on the original DBS, newborns with classic CAH have continued to be missed (3, 4).

A challenge of NBS for CAH is that biological factors influencing the hypothalamic-pituitary-adrenal (HPA) axis can cause 17OHP levels to be transiently higher or lower at 24 to 48 hours when the DBS sample is collected. Therefore, while FIA or LC-MS/MS may accurately capture 17OHP concentration levels at 24 to 48 hours of life, these levels may not accurately represent the presence or absence of disease in the newborn at the time of sample collection, as steroid assays cannot account for the inherent biological factors that influence HPA axis activity and 17OHP levels. Molecular testing, on the other hand, is not influenced by these biological factors or by timing of NBS specimen collection and may present a way to avoid the problems of steroid analysis. We hypothesized that *CYP21A2* molecular testing in conjunction with lowering the first-tier 17OHP cutoff level could potentially minimize FNs and improve overall CAH screening metrics.

## Materials and Methods

Our study involved 3 steps: the first 2 prospective and the third retrospective.

### Step 1

We created a Minnesota-specific pathogenic variant panel by identifying *CYP21A2* pathogenic variants specific to the Minnesota population to add to the panel of 12 common *CYP21A2* pathogenic variants used clinically for diagnosis. Blood samples were collected from Minnesota children with CAH and their parents at participating sites (University of Minnesota Masonic Children’s Hospital, the Mayo Clinic, and Children’s Hospitals of Minnesota). The Centers for Disease Control and Prevention (CDC) Newborn Screening and Molecular Biology Branch processed the blood samples to extract DNA, cryopreserve white blood cells, and create DBS. The study was approved by the institutional review board at all clinical sites. Informed consents from parents and assents from minors were obtained from all study participants. The CDC’s National Center for Environmental Health Office of Science determined that this work was not considered human subject research under 45 CFR 66.012(d) as all specimens were deidentified to the CDC.

**Figure 1.**
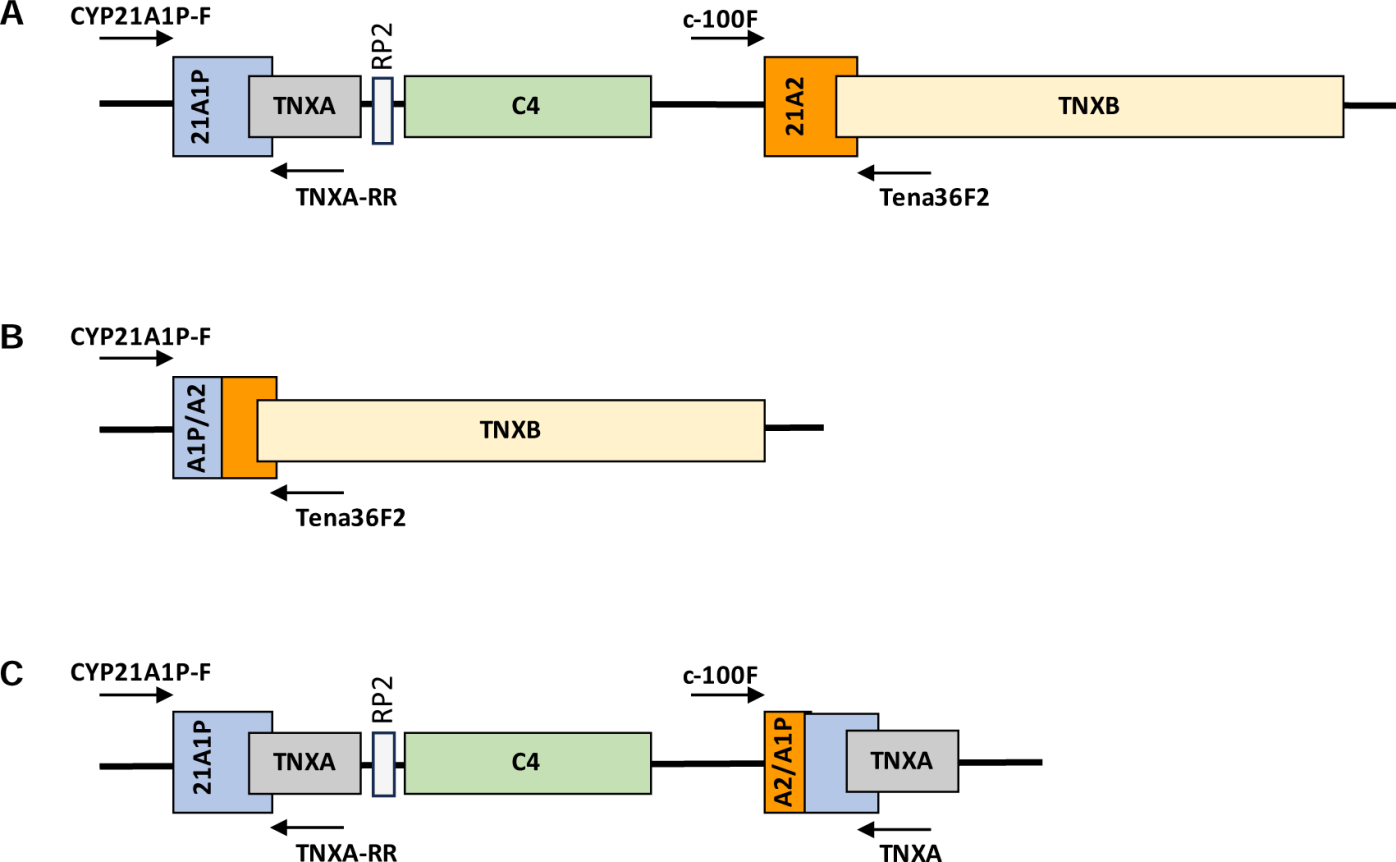
Long-range amplification-based method (LRAM) to detect A, the *CYP21A2* gene; B, 30-kb deletion; and C, large gene conversion alleles. A, The *CYP21A1P* pseudogene and *CYP21A2* gene are part of a 30-kb chromosomal duplication. B, Recombination between *CYP21A1P* and *CYP21A2* deletes a 30-kb section of the repeat forming a fusion of the 5′ end of the pseudogene with the 3′ portion of *CYP21A2*. C, A unidirectional transfer of pseudogene sequence to *CYP21A2* in a large-scale gene conversion results in a fusion of the 5′ end of *CYP21A2* with the 3′ end of *CYP21A1P*.

Disease-causing variants were detected using a long-range amplification-based method developed at the CDC (15) that amplified the *CYP21A2* gene as well as any recombinant 30-kb deletion or gene conversion alleles for each blood sample, and these amplicons were then detected by the Qiagen QIAxcel Capillary electrophoresis instrument and/or agarose gel electrophoresis (Fig. 1). All reactions contained an endogenous positive control to detect general assay failure. *CYP21A2* copy number was confirmed by a multiple ligation probe amplification (MLPA) SALSA MLPA CAH P050 kit from MRC Holland on all samples including samples negative for *CYP21A2* amplification and samples with apparent homozygous *CYP21A2* sequence to identify any potential hemizygosity. Amplicons were then Sanger-sequenced to detect variants in *CYP21A2* and the deletion breakpoints in any 30-kb deletion and gene conversion alleles, using the SeqScape software. Each allele’s predicted clinical phenotypes were determined by previous reports and publications, and phasing of multiple pathogenic variants in patient samples, cis vs trans, were inferred through inheritance patterns from parental samples. The novel variants’ potential pathogenicity was modeled using the amino acid substitution algorithms SIFT and PolyPhen2 (16).

### Step 2

A high-throughput allele-specific primer extension (ASPE) assay was developed at the CDC to detect the *CYP21A2* pathogenic variants identified in the Minnesota CAH population in addition to the core 12 *CYP21A2* pathogenic variants common to clinical testing panels. Following DNA extraction, the assay was accomplished in 2 parts: 1) long-range polymerase chain reaction (PCR) amplification of the *CYP21A2* gene and detection of the 30-kb deletion alleles, and 2) Luminex-based ASPE assay for the expanded Minnesota-specific panel of pathogenic variants. The ASPE assay was modeled after the assay developed by Oh et al (17) but modified to capture both large-scale gene conversions as well as small microgene conversions (intragenic conversions) in a single amplicon. Both assays were transferred from the CDC to the Minnesota Department of Health (MDH) where they were validated for in-house use.

Validation of the ASPE assay at CDC was performed using DBS samples from Minnesota families. The 30 kb deletion PCR and ASPE assays were validated through repeat testing from samples with previously known clinical genotypes. Allelic ratios for the ASPE genotypes were set through least 20 intrarun test results and 20 between-run tests, each repeated by 2 analysts, for each pathogenic and reference allele. ASPE primers, amplification primers, and sequence primers are listed in Supplementary Tables 1, 2, and 3 respectively (18).

### Step 3

**Figure 2.**
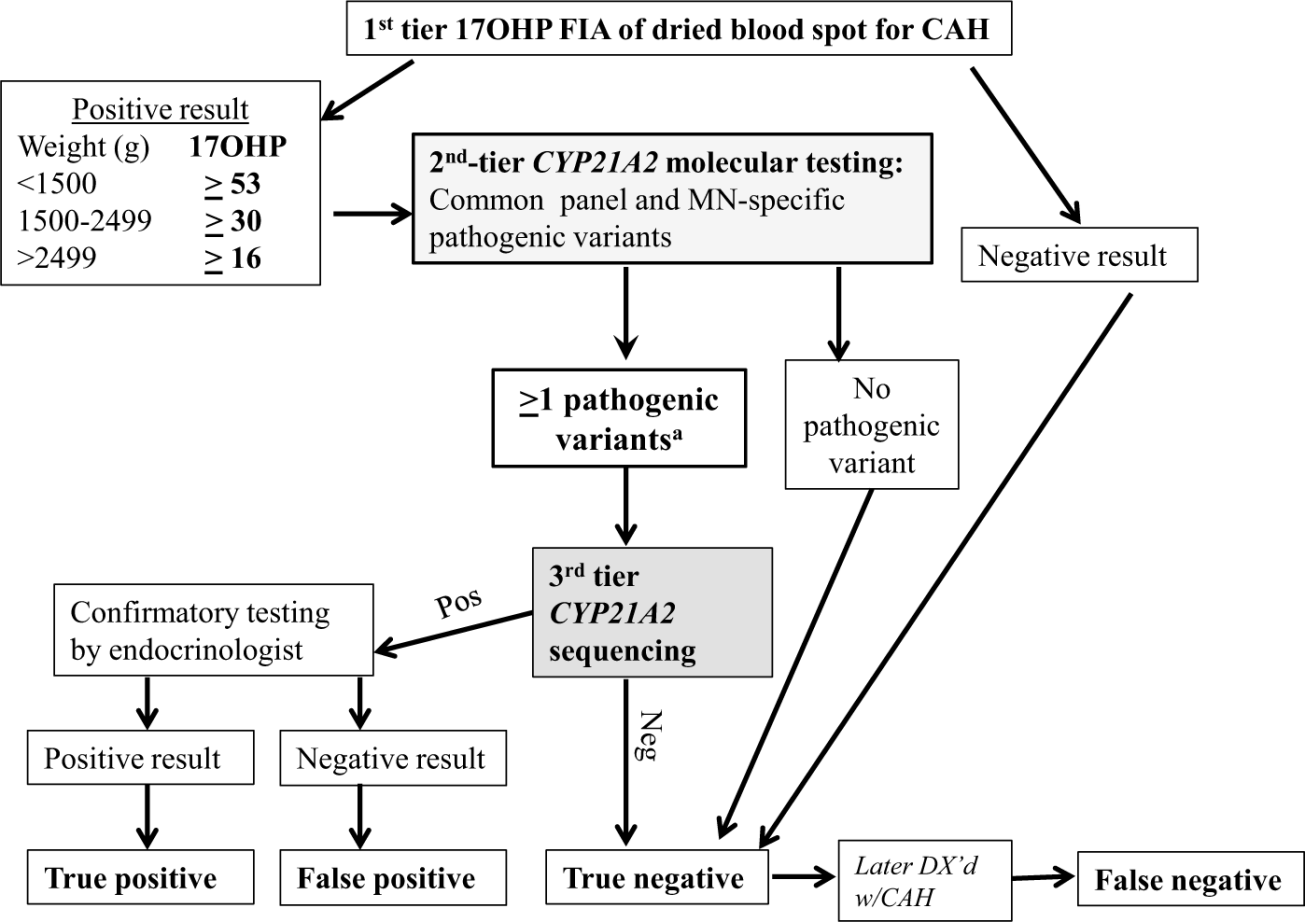
Algorithm of molecular-based protocol for newborn screening for congenital adrenal hyperplasia (CAH). A, Molecular protocol—all second-tier CYP21A2 samples with one or more pathogenic variants sent to third-tier sequencing. If 2 or more pathogenic variants are confirmed, the newborn was referred to pediatric endocrinologist for serum 17-hydroxyprogesterone (17OHP) and/or adrenocorticotropin stimulation confirmation testing.

**Figure 3.**
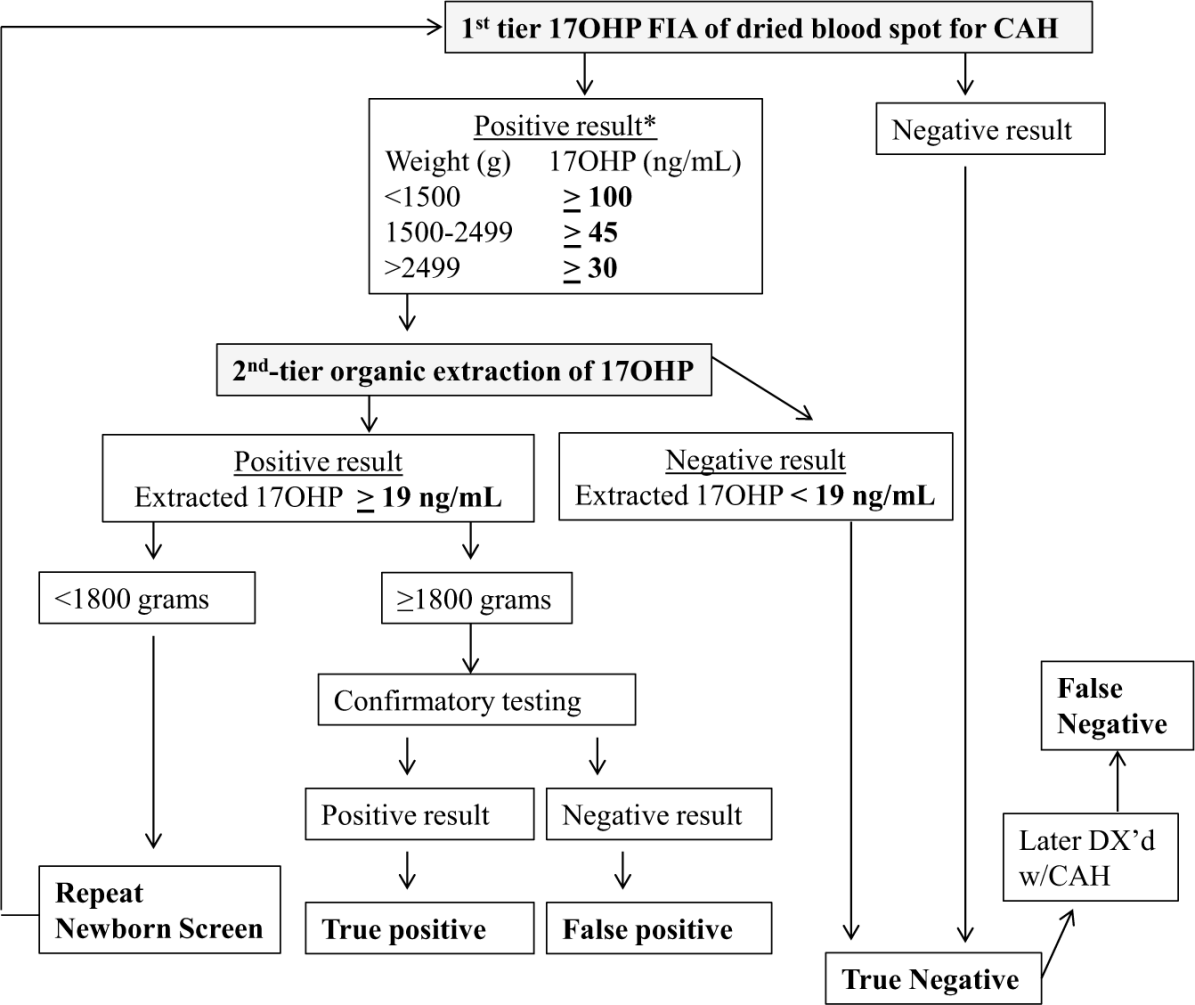
Algorithm of steroid-based protocol for newborn screening for congenital adrenal hyperplasia (CAH). *17-Hydroxyprogesterone (17OHP) value greater than or equal to 100 ng/mL and gestation age greater than 37 weeks are sent directly to confirmatory testing.

We performed a retrospective analysis of 1 year of NBS results generated by the MDH NBS program, comparing the metrics of the 2-tier steroid-based NBS protocol used by MDH that year vs a potential molecular-based NBS protocol using a lowered 17OHP cutoff. Both the molecular-based NBS protocols (Fig. 2) and steroid-based (Fig. 3) NBS protocols were assessed by examining DBS results from specimens received by MDH from January 1, 2015 to December 31, 2015. In all protocols first-tier 17OHP level, as measured by a PerkinElmer FIA assay performed on AutoDELFIA or Genetic Screening Processor (GSP) instrumentation, was assessed to determine whether second-tier testing was warranted. The steroid-based protocol used different weight-based 17OHP cutoff levels than the molecular-based protocols to trigger second-tier testing (Table 1).

For all protocols, the final diagnostic result after confirmatory testing was used to classify screening results for the infant as true positive (TP), true-negative (TN), or FP; in addition, those infants not referred for further testing were classified as TN. However, some infants classified as TN were subsequently reclassified as FN based on later, clinical diagnoses.

### Steroid-based newborn screening protocol

If the first-tier screening result was out of range, the original DBS was automatically sent for a second-tier diethyl ether extraction-based assay without contacting the newborn’s family (see Fig. 3). If the second-tier 17OHP level was normal, the result was reported as screen-negative. If the second-tier 17OHP level was elevated (≥19 ng/mL), the result was considered screen-positive and the newborn was referred to a pediatric endocrinologist for serum 17OHP and/or adrenocorticotropin stimulation confirmation testing to determine if the newborn was TP or FP.

In Minnesota, per the MDH 3-screen low birth weight steroid protocol, infants with a birth weight of less than 1800 grams had 3 17OHP FIA routine screens, the first collected at 24 to 48 hours, a second at 14 days, and a third at 30 days or at discharge. If any screen had elevated first-tier 17OHP, it was automatically sent for second-tier 17OHP testing. Confirmatory diagnostic testing was performed only if the third screen had elevated second-tier 17OHP or if the infant was symptomatic any time during the 3 screens.

### Molecular-based newborn screening protocol

Deidentified specimens received by the MDH from January 1, 2015 to December 31, 2015, were pulled for retrospective molecular analysis if the first-tier 17OHP FIA level was above the lowered 17OHP cutoff levels shown in Table 1. The lowered cutoff levels were based on the 17OHP levels of the known FN cases from 1999 through 2015, which resulted in the first-tier 17OHP cutoff levels being 33% to 47% lower than the steroid-based NBS protocol, depending on the weight category (3, 4). If the first-tier screening result was above the lowered 17OHP cutoff level, second-tier *CYP21A2* genotyping was performed on the original DBS (see Fig. 2) using the Minnesota-CDC panel of pathogenic variants. If no *CYP21A2* pathogenic variants were found, the result was considered screen-negative. If 1 or more pathogenic variants were found, the sample was sent for third-tier *CYP21A2* sequencing to validate the genotypes from the second-tier ASPE-genotyping assay. Samples confirmed to have 1 pathogenic variant were deemed TN. Samples confirmed to have 2 or more pathogenic variants were considered screen-positive, and the newborn would be referred to a pediatric endocrinologist for serum 17OHP and/or adrenocorticotropin stimulation confirmation testing to determine if the newborn was TP or FP (see Fig. 2).

### Statistical Methods

The final diagnostic result was used to classify the steroid-based NBS protocol and molecular-based NBS protocol result as TP, TN, FP, or FN. All screening metrics were calculated per each infant rather than per sample.

Separately for each birth-weight class and for all children combined, we compared the steroid-based and molecular-based NBS protocols according to these screening metrics:

- FN rate (FNR): FN/(TP + FN) = 1 – sensitivity
- PPV: TP/(TP + FP)
- FP rate (FPR): FP/(FP + TN) = 1 – specificity

The protocols were compared for FNR and FPR using a 2-sided exact McNemar test. For PPV, we used a permutation test because it is effectively exact and accounts for having applied the 3 NBS protocols to the same children. The permutation test was 2-sided, as follows: The test statistic was PPV for the molecular protocol minus PPV for the 2-tier steroid assay protocol; 10 000 artificial (ie, randomly permuted) data sets; for each such data set, the 2 protocol labels (steroid assay and molecular) were randomly permuted separately for each child. The *P* value is the fraction of the 10 000 artificial data sets in which the absolute value of the test statistic was greater than or equal to the absolute value of the test statistic in the actual data.

A secondary analysis examined the association between first-tier 17OHP concentrations (measured by FIA) and *CYP21A2* pathogenic variants grouped as “no pathogenic variant,” “1 pathogenic variant,” and “≥2 pathogenic variants.” Because the distribution of 17OHP concentration had an extremely long right tail, we considered the following tests: one-way analysis of variance (ANOVA) of the first-tier 17OHP concentrations, one-way ANOVA of the logarithm of the first-tier 17OHP concentrations (addressing the long tail), and the Wilcoxon rank-sum test (addressing the long tail and outliers).

## Results

*CYP21A2* gene sequencing of 103 Minnesota families (114 children affected with CAH, 249 total samples) identified an additional 9 rare, previously described, pathogenic variants and a novel predicted pathogenic intron 9 splice site variant. The CDC successfully designed ASPE probes for the 9 additional pathogenic variants, resulting in a final Minnesota-specific panel of 21 variants for the *CYP21A2* ASPE assay (Table 2). ASPE probe design failed for the Minnesota-specific p.Gly424Ser variant, and it was not included for the retrospective study. Overall, the combined carrier frequency for classic and NC *CYP21A2* pathogenic variants was 1 in 13 individuals.

The *CYP21A2* pathogenic variants and resulting genotypes identified by the ASPE and 30-kb deletion assays from DBS demonstrated 100% accuracy and reproducibility during the tests when compared to the genotypes identified by *CYP21A2* gene sequencing of the respective blood samples. The ASPE assay was transferred from the CDC to the MDH. Adjustments were made by the MDH to the workflow process including a modification of the DNA extraction procedure, so that the genotyping procedure could be completed in a single day.

The NBS results and metrics of the steroid-based and molecular-based NBS protocols are listed in Tables 3 and 4. In 2015, a total of 68 659 live births in Minnesota were screened by the MDH NBS program. Of these 68 659 children, 515 did not have recorded birth weights, all of whom were TN for CAH at the time of this report and tested negative under both the steroid-based and molecular-based NBS protocols. These 515 children were allocated to birth weight classes (6 to <1500 g, 28 to 1500–2499 g, and 481 to ≥ 2500 g) in proportion to the counts in each birth-weight class among the 68 144 children who had recorded birth weights (821 < 1500 g, 3685 1500–2499 g, and 63 638 ≥ 2500 g). Also, 6 NBS samples gave unsatisfactory results on the molecular CAH protocol on the initial run and rerun; these 6 children were included in Table 3 under the extracted 17OHP second-tier steroid NBS protocol but excluded from Table 3 under the molecular NBS protocol and excluded from analyses comparing the steroid and molecular protocols.

The molecular and steroid-based NBS protocols applied different weight-based 17OHP cutoff levels to the first-tier 17OHP FIA results to determine which samples would be sent for second-tier testing (see Table 1). The final outcomes of the steroid-based NBS protocol used by the MDH over the course of 1 year include 2 FNs, 68 565 TNs, 91 FPs, and 1 TP (see Table 3), for a PPV of 1.1% and an FPR of 0.13% (see Table 4).

The molecular NBS protocol’s lower 17OHP FIA first-tier cutoff level resulted in 4018 out-of-range first-tier results compared to 621 out-of-range results for the steroid-based NBS protocol that used the original higher first-tier cutoff levels used by the MDH NBS program in 2015. Per the molecular protocol, 4018 results were automatically sent for second-tier *CYP21A2* genotyping, which identified 55 samples with 2 or more pathogenic variants and 309 with a single pathogenic variant. None of the 309 infants with a single pathogenic variant were found to have any additional pathogenic variants after third-tier *CYP21A2* sequencing. Overall, applying the molecular protocol would have resulted in 0 FNs, 68 585 TNs, 52 FPs, and 3 TPs, for a PPV of 5.45% and an FPR of 0.08% (see Table 4).

Specifics of the 2 FN cases missed under the steroid-based NBS protocol were as follows: In case 1, the female newborn’s first-tier 17OHP by FIA concentration was 28.8 ng/mL, below the steroid based NBS protocol cutoff level (≥30 ng/mL) used for infants weighing more than 2500 g (4179 g), so second-tier steroid testing was not triggered (see Fig. 3). Under the molecular-based NBS protocol, the first-tier 17OHP was above the cutoff level (>16 ng/mL), triggering second-tier molecular testing, which identified 2 *CYP21A2* pathogenic variants (p.Ile172Asn/IVS2–13 G) (see Fig. 2). Case 1 was diagnosed with SV-CAH when she presented with genital virilization at age 10 months. When the FN was reported, the MDH-NBS program pulled the original DBS and measured the 17OHP level using the second-tier diethyl ether extraction assay. The result was within normal range (9.7 ng/mL, ≥ 19.0), suggesting that the newborn would not have been identified with CAH even if her first-tier sample was measured by the second-tier steroid assay.

In case 2, the female newborn’s (birth weight 3152 g) first-tier 17OHP by FIA level was elevated at 37.7 ng/mL (≥30 ng/mL), which automatically triggered second-tier testing of the original DBS. The second-tier diethyl ether extraction assay reported a 17OHP level of 12.5 ng/mL, below the cutoff (≥19.0), and therefore the result was deemed screen-negative and the newborn was not referred for confirmatory testing. Ultimately, case 2 was diagnosed with SV-CAH when she presented with genital virilization at age 2.5 years. In the molecular NBS protocol, case 2’s elevated first-tier 17OHP level would have triggered second-tier molecular testing, which identified 2 CYP21A2 pathogenic variants (p.Ile172Asn/p.Ile172Asn).

In a secondary analysis of first-tier 17OHP concentrations (measured by FIA) in the 3648 specimens with no *CYP21A2* pathogenic variants, 309 specimens in the “1 pathogenic variant” group, and 55 specimens in the “≥2 pathogenic variants” group found 1 outlier in each of the latter 2 groups: 596 and 635 ng/mL in “1 pathogenic variant” and “≥2 pathogenic variants” respectively, compared to the second-largest levels of 209 and 155 ng/mL, respectively. The median 17OHP for both groups was 21 ng/mL. The 17OHP concentration was significantly different between groups using 1-way ANOVA (*P* = .0004); however, when the 2 outliers were excluded from the 1-way ANOVA, or 1-way ANOVA was applied to the logarithms of 17OHP concentration, or Wilcoxon nonparametric test was used, the differences were insignificant, with *P* values of .77, .33, and .81, respectively (Supplementary Fig. 1 depicts first-tier 17OHP concentrations of NBS specimens with no pathogenic variants vs 1 pathogenic variant vs 2 or more pathogenic variants) (18).

## Discussion

To ensure timely identification of all newborn disorders, it is recommended that DBS specimens be collected 24 to 48 hours after birth (19). Early collection of samples creates a conundrum for state programs that use steroid assays for CAH NBS because even if the steroid assay accurately measures a newborn’s 17OHP level at the collection time, 17OHP levels can be either transiently high at birth in unaffected newborns, particularly premature infants (5), or transiently low in affected newborns.

This is the first report in the United States of a CDC-developed, rapid, high-throughput multiplex ASPE second-tier NBS assay that was used to examine the utility of molecular testing in NBS for CAH. Comparing the 2-tier steroid-based NBS protocol used by the MDH in 2015 with the 3-tier molecular-based NBS protocol, we found the molecular-based protocol would result in improved FNs and FPs (see Tables 3 and 4 and Figs. 2 and 3).

### False Negatives

In recent years, reported FNR rates in CAH newborn screening vary among state NBS programs in the United States. Although some programs report no FNs, the accuracy of reported rates is susceptible to unreported cases of classic CAH that get diagnosed months or years after the original screen and depend on the diagnosing physician reporting the missed case to their state NBS program.

Biological factors can lead to FNs in steroid-based assays applied to specimens collected soon after birth. Transiently low 17OHP levels in a CAH-affected infant can be due to suppression of the fetal HPA axis activity in utero either by increased sensitivity of the fetal HPA axis to maternal cortisol or by increased maternal cortisol levels in fetal circulation due to decreased inactivation of maternal cortisol to cortisone by the placental enzyme 11β-hydroxysteroid dehydrogenase type II (11β-HSD-2).

During pregnancy most maternal cortisol cannot normally reach the fetus because it is oxidized by the placental enzyme 11β-HSD-2 to cortisone, the inactive form of cortisol (20, 21). Downregulation of the *11β-HSD-2* gene may alter the enzyme activity, allowing a greater proportion of maternal cortisol to cross into the placenta and then enter fetal circulation. Also, by 34 to 35 weeks’ gestation, activity of placental 11β-HSD2 decreases, which in turn allows a surge of maternal cortisol into fetal compartments (22, 23). In a fetus affected with CAH, a decrease in 11β-HSD-2 activity would allow higher amounts of maternal cortisol in fetal circulation, leading to decreased activity of the fetal HPA axis and transiently low 17OHP levels at 24 to 48 hours after birth when the DBS sample is collected. The later the timing of the collection of an NBS or serum confirmation sample from affected infants, the higher the 17OHP concentration (3–5). Another potential cause of transiently low 17OHP is increased glucocorticoid sensitivity to maternal cortisol that crosses into fetal circulation. Specific glucocorticoid receptor haplotypes have been associated with varying glucocorticoid sensitivity, including those that cause individuals to be more sensitive to glucocorticoids (24, 25).

In the present study, both cases of CAH-affected newborns missed under the steroid-based NBS protocol illuminate the shortcomings of steroid-based first-tier and second-tier assays. Steroid-based assays, even the most sensitive, cannot account for the influence of factors during intrauterine life, fetal HPA axis glucocorticoid sensitivity, or the state of the HPA axis at the time of the NBS collection. In case 1, the first-tier 17OHP by FIA level was below the steroid-based NBS protocol cutoff level so that second-tier steroid testing was not triggered and it was reported as a TN (see Fig. 3). In contrast, the 17OHP level was above the molecular NBS protocol’s cutoff level and would have triggered second-tier molecular testing that identified 2 *CYP21A2* pathogenic variants. In case 2, the first-tier 17OHP level was elevated (screen-positive) and automatically triggered second-tier testing. However, the second-tier diethyl ether extraction assay reported the 17OHP level as normal; therefore, the result was deemed screen-negative and the newborn was not referred for confirmatory testing. Specimens of newborns affected by CAH correctly identified to be out of range by first-tier FIA but ultimately negated by normal second-tier results have been reported (3, 4). In our previous study of NBS for CAH in Minnesota, second-tier testing by LC-MS/MS overturned 4 cases with out-of-range first-tier results, leading to 4 FN cases (3). Under the molecular NBS protocol, case 2’s elevated first-tier 17OHP level would have triggered second-tier molecular testing, which identified 2 *CYP21A2* pathogenic variants.

In addition to the risk of adrenal and/or SW crisis, late diagnoses due to missed cases of CAH can harm children’s health outcomes, including growth and pubertal development. As we previously reported, 5 male children with SV-CAH missed by Minnesota NBS in the years 1999 to 2010 and not diagnosed until ages 2.3 to 5.5 years had significant bone age advancement (median 7 years difference between bone age and chronological age) and required multiple costly interventions to address precocious puberty, advanced bone age, and reduced predicted final adult height (4). All 5 had increased emergency room visits or hospitalizations before diagnosis due to protracted symptoms with acute illness. During the same period, 3 female children with SV-CAH missed by NBS had atypical genitalia at birth, but were not diagnosed until months or years later (3 months, 3.4 years, and 6.5 years) (3). Two of these female patients also had evidence of SW and were started on fludrocortisone.

A potential solution to address FNs in steroid-based protocols would be to lower the 17OHP cutoff levels of first-tier FIA to correspond to the lowest levels of known FN cases within a state’s NBS program as was done in our study for the molecular protocol. Hypothetically, if the MDH lowered the steroid-based protocol’s 17OHP cutoff levels to the same as the molecular protocol, it would have generated 4018 first-tier screen-positives that would be sent to a second-tier diethyl ether extraction method rather than the 621 first-tier samples that were actually sent that year. Using the same percentage of FPs that occurred under the steroid-based protocol would have led to approximately 595 FPs in this hypothetical example, a significantly higher number than would be acceptable.

### False Positives

FPs have long presented a challenge to NBS for CAH. Levels of 17OHP can be transiently high at birth, particularly in premature newborns (5, 26). During the first 2 weeks of life in preterm infants, 17OHP levels have been shown to be inversely correlated with postconceptional age, with the normalization of 17OHP concentrations being slower in infants with birth weight of less than 1000 g and gestational age between 26 and 28 weeks (27). Several confounding biological factors associated with prematurity such as physiologically lower enzymatic activity of the 21-hydroxylase and/or 11β-hydroxylase enzymes due to immaturity of the adrenal glands, birth stress, illness, and poor kidney function can cause transiently elevated 17OHP levels and high FPRs in NBS for CAH in premature babies (28–30). We have previously reported that later sample collection in premature babies (<1800 g) plays a more important role in reducing FP results than the use of a second-tier steroid-assay (5). In the present report (see Tables 3 and 4), most FPs for the steroid-based NBS protocol were seen in newborns weighing less than 2500 (76/91) FPs. In contrast, the molecular protocol resulted in 52 FPs with only 5 in newborns weighing less than 2500 g. The reason for the significantly higher number of FPs in newborns weighing less than 2500 g is that first- and second-tier steroid-based assays are time dependent in that they capture 17OHP concentrations at the time of sample collection, which can be transiently high thus leading to more FPs. Steroid-based assay’s time dependency was the impetus for the 3-screen low birth weight protocol (collections at 24–48 hours, at 2 weeks, and at 30 days) as it allows for the HPA axis to recover. The reason for fewer FPs in less than 2500-g newborns in the molecular protocol is because second-tier molecular screening results are not affected by the immaturity of the HPA axis. As a result NBS samples with transiently elevated 17OHP levels are less likely to have multiple pathogenic variants and therefore second-tier molecular testing will lead to fewer FPs in newborns weighing less than 2500 g compared to those weighing more than 2500 g.

### Challenges and Limitations of Molecular Newborn Screening for Congenital Adrenal Hyperplasia

Families and individuals commonly have multiple *CYP21A2* variants in *cis* configuration (ie, on the same parental chromosome), which presents a challenge for interpreting and reporting test results. As part of testing the 103 families, we found that 13% of the CAH families in this study had 2 or more *CYP21A2* variants on the same parental chromosome as determined by the inheritance patterns of the parents to the affected probands.

Since parental blood samples were not available for the 1-year retrospective analysis of NBS specimens, it could not be determined if multiple pathogenic *CYP21A2* variants were in *cis*, indicating a carrier state, or in *trans*, indicating a possible CAH disease state. Future research on improving the identification of the phase of detected pathogenic variant without the need for parental blood samples may help elucidate a molecular protocol that could eliminate both FPs and FNs while maintaining specificity. Using the Minnesota family samples with 2 or more pathogenic *CYP21A2* variants and known phasing, Greene et al (31) showed that manual determination of phasing by using an allele-specific PCR and a total of 5 variant sites can improve assessment of CAH disease risk in a newborn without the requirement of family studies for genetic phasing. Until new sequencing techniques allow phasing of the pathogenic variants in the NBS samples newborn without the requirement of family studies, it would be timelier to refer all newborns with 2 or more pathogenic variants found by second-tier directly for confirmatory testing and skip third-tier sequencing.

Lowering the first-tier 17OHP cutoff level could present another challenge. When the first-tier 17OHP cutoff levels in the molecular NBS protocols were set to the lowest known 17OHP levels of known FN cases in Minnesota from 1999 through 2015, 4018 specimens were reflexed to second-tier *CYP21A2* genotyping whereas 621 specimens were reflexed to second-tier using the steroid-based protocol’s higher first-tier 17OHP cutoff levels (see Table 1). If the 17OHP cutoff level of the molecular protocol was set higher at 18 ng/mL instead of 16 ng/mL, the number of samples reflexed to second-tier *CYP21A2* genotyping would have dropped to 3191 and we still would have identified the 2 FN samples missed by the steroid-based protocol.

Another challenge in implementing a molecular NBS protocol is the time it takes to report the final result. In most cases the timeline for an NBS program to complete the 3 tiers of the molecular NBS protocols would be 5 days. However, various factors could affect the timeline (eg, batch analyzing, number of personnel, equipment) and the process could take up to 7 days, which would still be within a comparable timeline seen in states requiring 2 screens. Currently 13 states require a routine second screen from all infants between 8 and 14 days of life, which improves NBS metrics because it allows the transient levels to reflect disease state. A recent report from the Northwest Regional Newborn Screening Program found that 25% of affected infants were identified on the second screen, of whom 39% were diagnosed with SW-CAH (32).

One limitation of this study is that retrospectively performing the second-tier *CYP21A2* genotyping and third-tier *CYP21A2* sequencing of 1 year’s NBS specimens does not allow reporting of potential confounding factors that may be identified when performing the second-tier and third-tier tests live. Also, while the study’s retrospective nature allowed identification of cases of CAH missed by NBS, it is still possible that other children with CAH were not yet diagnosed or had moved to another state. A prospective, multi-state study incorporating molecular testing into current protocols would be needed to assess metrics, costs, and confounding factors.

Another study limitation was the use of a single year of NBS data. The numbers of FP and FN results for CAH fluctuates from year to year, as does the PPV, which in surrounding years varied in a range from 1% to 4%. That range is low compared with the roughly 8% PPV previously reported for 2-tier screening for CAH in Minnesota using a second-tier LC-MS/MS assay (12).

In conclusion, although introducing molecular testing into NBS protocols significantly improved the overall NBS for CAH metrics, a state NBS program would need to consider many factors as molecular testing in the NBS for CAH poses its own unique challenges.

## Supplementary Material

Table 2 - Amplification Primers

Table 1 - ASPE Primers

Figure 1 - 17OHP Values

Table 3 - Sequencing primers

## Figures and Tables

**Table 1.** 17-Hydroxyprogesterone weight-based cutoff levels for first-tier fluoroimmunoassay and second-tier diethyl ether extraction based assay in the newborn screening for congenital adrenal hyperplasia

**1st tier 17OHP by FIA**		

Weight, g	Molecular protocol 17OHP cutoff, ng/mL^a^	Steroid-based protocol 17OHP cutoff, ng/mL^b^
<1500	≥53	≥100
1500-2499	≥30	≥45
≥2500	≥16	≥30

**Second-tier 17OHP by diethyl ether extraction**		

Not weight based	N/A^c^	≥19.0

Abbreviations: 17OHP, 17-hydroxyprogesterone; FIA, fluoroimmunoassay; N/A, not available; NBS, newborn screening.

a The molecular protocol used lower first-tier 17OHP FIA cutoff levels based on the 17OHP values of known false negatives in Minnesota since 1999.

b First-tier 17OHP FIA cutoff levels used by the Minnesota Department of Health in 2015.

c Second tier for the molecular NBS protocol was performed by molecular (allele-specific primer extension) assay.

**Table 2.** CYP21A2 allele-specific primer extension assay genotyping panel

Common diagnostic panel	Gene position	Allele based on ref sequence NM_00500.6	Predicted clinical manifestation

p.Pro30Leu	Exon 1	c.92C > T	NC-SV
IVS2-13 A/C > G	Intron 2	c.293-13A/C > G	SV-SW
p.Gly110del8	Exon 3	c.332_339del8	SW
p.Ile172Asn	Exon 4	c.518T > A	SV
p.Ile236Asn	Exon 6	c.710T > A	SW
p.Val237Glu	Exon 6	c.713T > A	SW
p.Met239Lys	Exon 6	c.719T > A	SW
p.Val281Leu	Exon 7	c.844G > T	NC
p.Leu307fs	Exon 7	c.921Ins1	SW
p.Gln318X	Exon 8	c.955C > T	SW
p.Arg356Trp	Exon 10	c.1069C > T	SW
p.Pro453Ser	Exon 10	c.1360C > T	NC

**Additional Minnesota patient pathogenic variants**	**Gene position**	**Allele based on ref sequence NM_00500.6**	**Predicted clinical manifestation**

p.His62Leu	Exon 1	c.185A > T	NC-SV
p.Arg149Cys	Exon 4	c.445C > T	NC
p.Leu307Val	Exon 7	c.922T > G	SW
p.His365Tyr	Exon 8	c.1096C > T	SW
IVS9 + 1 G > T	Intron 9	c.1219 + 1G > T	Predicted SW
p.Arg426Cys	Exon 10	c.1276C > T	SW
p.Arg426Pro	Exon 10	c.1277G > C	SV
p.Arg444X	Exon 10	c.1330C > T	SW
p.Arg483fs	Exon 10	c.1448_1449delGGinsC c.1448_1449delGG	SW

Abbreviations: NC, not classic; SV, simple virilizing; SW, salt-wasting.

**Table 3.** Newborn screening results of the molecular-based protocol and the steroid-based protocol over 1 year period

No. of infants screened (n = 68 659)	Molecular NBS protocol^a^				Steroid NBS protocol^b^			

Birth weight, g	**<1500**	**1500–2499**	**≥2500**	**Total**	**<1500**	**1500–2499**	**≥2500**	**Total**
1st-tier 17OHP FIA cutoff, ng/mL	≥53	≥30	≥16		≥100	≥45	≥30	
1st-tier out-of-range results requiring 2nd-tier testing	230	522	3266	**4018**	38	161	422	**621**
2nd-tier screen-positive cases with elevated 17OHP by diethyl ether extraction sent to Ped Endo for confirmatory testing^c^	—	—	—	—	37	39	16	**92**
2nd-tier screen-positive cases with ≥1 pathogenic variants sent to 3rd-tier sequencing^a^	26	35	303	**364**	—	—	—	—
TP results	0	0	3	**3** ^c^	0	0	1	**1** ^c^
FP results	2	3	47	**52** ^c^	37	39	15	**91** ^c^
FN results	0	0	0	**0**	0	0	2	**2**
TN results	824	3709	64 065	**68 598**	790	3674	64 101	**68 565**

The total number of infants screened from live births in 2015 was 68 659. The molecular protocol’s first-tier 17OHP FIA cutoff levels were 47%, 33%, and 47% lower than the steroid-based protocol in the less than 1500 g, 1500 to 2499 g, and 2500 g or greater birth weights, respectively. Values are counts of infants, or rates. Second-tier unsatisfactory specimens in the molecular NBS protocol (n = 6) and in the steroid-based protocol (n = 75). Birth weight was not recorded at original hospital for 515 newborns, all of whom were identified as negative on first tier; for purpose of analyses these newborns were assigned a birth weight using the same fractions as the 68 144 newborns with weights.

Abbreviations: 17OHP, 17-hydroxyprogesterone; FIA, fluoroimmunoassay; FP, false positive; FN, false negative; NBS, newborn screening; TN, true negative; TP, true positive.

a Molecular-based NBS protocol A: 1st-tier 17OHP FIA; 2nd-tier molecular assay (all specimens with ≥1 *CYP21A2* pathogenic variants automatically sent to 3rd tier); 3rd-tier *CYP21A2* sequencing.

b Steroid-based NBS protocol: 1st-tier 17OHP FIA; 2nd-tier diethyl ether extraction-based assay.

c Under the molecular-based NBS protocol, 55 infants with 2 or more pathogenic mutations would have been referred to a pediatric endocrinologist for confirmation testing; Under the steroid NBS protocol, 92 would have been referred for confirmation testing.

**Table 4.** Comparison of newborn screening (NBS) metrics between the molecular-based NBS protocol vs steroid-based NBS protocol over 1 year period

All newborns screened	Molecular NBS protocol^a^	Steroid-based NBS protocol^b^	Molecular vs steroid *P*

Sensitivity, %	100	33.3	***P* = .50**
Specificity, %	99.924	99.867	***P* = .0013**
Positive predictive value, %	5.45	1.09	***P* = .0005**
FN rate, %	0	66.7	***P* = .50**
FP rate, %	0.076	0.133	***P* = .0013**
**Birth weight <2500 g**			
FP rate, %	0.088	1.674	***P* < .0001**
**Birth weight >2500 g**			
FP rate, %	0.0749	0.0234	***P* < .0001**

Sensitivity (%) = TP/(TP + FN)×100; specificity (%) = TN/(FP + TN)×100; positive predictive value (%) = TP/(TP + FP)×100; FN rate (%) = FN/(FN + TP)×100 = 100 – sensitivity (%); FP rate (%) = FP/(FP + TN)×100 = 100 – specificity (%). Second-tier unsatisfactory specimens in the molecular NBS protocol (n = 6) and in the steroid-based protocol (n = 75).

Abbreviations: 17OHP, 17-hydroxyprogesterone; FIA, fluoroimmunoassay; FP, false positive; FN, false negative; NBS, newborn screening; TN, true negative; TP, true positive.

a Molecular-based NBS protocol: first-tier 17OHP FIA; second-tier molecular assay (all specimens with ≥1 *CYP21A2* pathogenic variants automatically sent to third-tier *CYP21A2* sequencing).

b Steroid-based NBS protocol: first-tier 17OHP FIA; 2nd-tier diethyl ether extraction-based assay.

## Data Availability

Restrictions apply to the availability of some or all data generated or analyzed during this study to preserve patient confidentiality or because they were used under license. The corresponding author will on request detail the restrictions and any conditions under which access to some data may be provided.

## Abbreviations:

11β-HSD-2: 11β-hydroxysteroid dehydrogenase type II
17OHP: 17-hydroxyprogesterone
ANOVA: analysis of variance
ASPE: allele-specific primer extension
CAH: congenital adrenal hyperplasia
CDC: Centers for Disease Control and Prevention
DBS: dried blood spot
FIA: fluoroimmunoassay
FP: false positive
FPR: false-positive rate
FN: false negative
HPA: hypothalamic-pituitary-adrenal
LC-MS/MS: liquid chromatography–tandem mass spectrometry
MDH: Minnesota Department of Health
NBS: newborn screening
NC: not classic
PCR: polymerase chain reaction
PPV: positive predictive value
TN: true negative
TP: true positive
SV: simple virilizing
SW: salt-wasting
