# Supplementary material for: Can Incorporating Molecular Testing Improve the Accuracy of Newborn Screening for Congenital Adrenal Hyperplasia?
Source: J Clin Endocrinol Metab. Author manuscript; Available in PMC 2025 Mar 18. (PMC11531606; doi:10.1210/clinem/dgae297)
Supplement: Supplementary file 1 [file NIHMS2012880-supplement-Figure_1_-_17OHP_Values.docx]

**Supplemental Figure 1. MDH 17OHP Value (ng/mL) vs. N mutations in molecular test**

**Percentiles**

| **Group** | **Minimum** | **10%** | **25%** | **Median** | **75%** | **90%** | **Maximum** |
| --- | --- | --- | --- | --- | --- | --- | --- |
| A: 0 mutations | 16 | 16.6 | 17.9 | 21.2 | 32.1 | 48.3 | 388.9 |
| B: 1 mutation | 16 | 16.6 | 17.8 | 21 | 31.65 | 58.6 | 596.1 |
| C: ≥2 mutations | 16.2 | 16.79 | 17.8 | 20.85 | 34.15 | 55.54 | 634.6 |
