# Supplementary material for: Can Incorporating Molecular Testing Improve the Accuracy of Newborn Screening for Congenital Adrenal Hyperplasia?
Source: J Clin Endocrinol Metab. Author manuscript; Available in PMC 2025 Mar 18. (PMC11531606; doi:10.1210/clinem/dgae297)
Supplement: Supplementary file 2 [file NIHMS2012880-supplement-Table_1_-_ASPE_Primers.docx]

**Supplemental Table 1.  ASPE Primers**

| ***CYP21A2*** **Variant*** | **ASPE Probe Sequence**** | **mTAG Beadset** | **Allele and Phenotype** |
| --- | --- | --- | --- |
| **p.Pro30Leu_C** | TAATCCGGAGCCTCCACCTCCC | 14 | Normal |
| **p.Pro30Leu_T** | TAATCCGGAGCCTCCACCTCCT | 12 | Variant - NC |
| **p.His62Leu_A** | GCCCATCTACAGGCTCCA | 37 | Normal |
| **p.His62Leu_T** | GCCCATCTACAGGCTCCT | 39 | Variant - NC |
| **c.290-13_A (IVS2-13_A)** | CCCACCCTCCAGCCCCCAA | 18 | Normal |
| **c.290-13_C (IVS2-13_C)** | CCCACCCTCCAGCCCCCAC | 20 | Normal |
| **c.290-13_G (IVS2-13_G)** | CCCACCCTCCAGCCCCCAG | 22 | Variant – SV/SW |
| **p.Gly110del8_A** | CCGGACCTGTCCTTGGGA | 28 | Normal |
| **p.Gly110del8_del** | CCGGACCTGTCCTTGGT | 26 | Variant - SW |
| **p.Arg149Cys_C** | CCTTCGGCTGCGGTGCCC | 57 | Normal |
| **p.Arg149Cys_T** | CCTTCGGCTGCGGTGCCT | 62 | Variant - NC |
| **p.Ile172Asn_A** | CTCTCCTCACCTGCAGCATCAA | 30 | Variant - SV |
| **p.Ile172Asn_T** | CTCTCCTCACCTGCAGCATCAT | 34 | Normal |
| **p.Ile236Asn_A** | CCATAGAGAAGAGGGAYCACAA | 36 | Variant - SW |
| **p.Ile236Asn_T** | CCATAGAGAAGAGGGAYCACAT | 38 | Normal |
| **p.Val237Glu_A** | GAAGAGGGATCACATSGA | 67 | Variant - SW |
| **p.Val237Glu_T** | GAAGAGGGATCACATSGT | 73 | Normal |
| **p.Met239Val_A** | GGAYCACAWSGWGGAGAA | 75 | Variant – No Disease |
| **p.Met239Val_T** | GGATCACATSGTGGAGAT | 77 | Normal |
| **p.Val281Leu_G** | CAGCTCCTGGAAGGGCACG | 44 | Normal |
| **p.Val281Leu_T** | CAGCTCCTGGAAGGGCACT | 42 | Variant - NC |
| **p.Leu307fs_G** | CACCCTCTCCTGGGCCGTGGTTTTTTTG | 46 | Normal |
| **p.Leu307fs_insT** | CACCCTCTCCTGGGCCGTGGTTTTTTTT | 48 | Variant - SW |
| **p.Leu307Val_G** | CACCCTCTCCTGGGCCGTGGTTTTTG | 13 | Variant - NC |
| **p.Gln318X_C** | CCCAGATTCAGCAGCGACTGC | 54 | Normal |
| **p.Gln318X_T** | CCCAGATTCAGCAGCGACTGT | 52 | Variant - SW |
| **p.Arg365Trp_C** | GCCGAGGTGCTGCGCCTGC | 61 | Normal |
| **p.Arg365Trp_T** | GCCGAGGTGCTGCGCCTGT | 56 | Variant - SW |
| **p.His365Tyr_C** | TGCCCTTAGCCTTGCCCC | 15 | Normal |
| **p.His365Tyr_T** | TGCCCTTAGCCTTGCCCT | 19 | Variant – SV/SW |
| **c.1219+1_G** | ACATGAGTTCTGGCCTGG | 43 | Normal |
| **c.1219+1_T** | ACATGAGTTCTGGCCTGT | 45 | Variant - SW |
| **p.Arg426Cys_C** | CCTTCGGCTGCGGTGCCC | 25 | Normal |
| **p.Arg426Cys_T** | CCTTCGGCTGCGGTGCCT | 27 | Variant - SW |
| **p.Arg426Pro_C** | CCTTCGGCTGCRGTGCCYC | 55 | Variant - SW |
| **p.Arg426Pro_G** | ACACTGTTTCTCCACAGC | 53 | Normal |
| **p.Arg444X_C** | TCTTCGTGGTGCTGACCC | 29 | Normal |
| **p.Arg444X_T** | TCTTCGTGGTGCTGACCT | 21 | Variant - SW |
| **p.Arg453Ser_C** | GCAGGCCTTCACGCTGCTGC | 65 | Normal |
| **p.Pro453Ser_T** | GCAGGCCTTCACGCTGCTGT | 63 | Variant - NC |
| **p.Pro483fs_G** | CGGCTGCAGCCCCGGGGG | 33 | Normal |
| **p.Arg483fs_delGGinsC/delGG** | CGGCTGCAGCCCCCGGGA | 35 | Variant - SW |

SW, salt-wasting; SV, simple-virilizing

*Allele designation based on Reference Sequence NM_00500.6

**Complementary sequence to Luminex mTAG nucleotide tags not shown
