# Supplementary material for: Can Incorporating Molecular Testing Improve the Accuracy of Newborn Screening for Congenital Adrenal Hyperplasia?
Source: J Clin Endocrinol Metab. Author manuscript; Available in PMC 2025 Mar 18. (PMC11531606; doi:10.1210/clinem/dgae297)
Supplement: Supplementary file 3 [file NIHMS2012880-supplement-Table_2_-_Amplification_Primers.docx]

**Supplemental Table 2. Amplification Primers**

| **Primer** | **Function** | **Sequence** | **Site** |
| --- | --- | --- | --- |
| c-100F | CYP21A2 & gene conversion forward | TTTCCCTTCCTTGCTTCTTGATGGGTGA*T*C | CYP21A2 Promoter |
| Tena36F2 | CYP21A2 & 30kb deletion reverse | AGGCGCTCGCTATGAGGTGAC | TNXB 3' UTR |
| CYP21A1P-F (pt) | CYP21A1P & 30kb deletion forward | CTGAGTTCTGTGAAATTCCCCAATCCTTACTTTT*T*G | CYP21A1P Promoter |
| TNXA-R | CYP21A1P & gene conversion reverse | GGACACAGAAACTCCAGGTGGGATT | TNXA 3' UTR |
| ComUTR | CYP21A2 & gene conversion reverse | AGCACAGTGGACCATCAGGT | CYP21A2 3' UTR |
| Internal positive amplification control - AVPR2 |  |  |  |
| ME0004 | Control R | ACCTGGCCGTGGCTCTGTTC | AVPR2 upstream primer |
| ME0027 | Control F | ACAGGCTCTGGCCAATTCTC | AVPR2 downstream primer |

*Phosphorothioate linkage
