# Supplementary material for: Can Incorporating Molecular Testing Improve the Accuracy of Newborn Screening for Congenital Adrenal Hyperplasia?
Source: J Clin Endocrinol Metab. Author manuscript; Available in PMC 2025 Mar 18. (PMC11531606; doi:10.1210/clinem/dgae297)
Supplement: Supplementary file 4 [file NIHMS2012880-supplement-Table_3_-_Sequencing_primers.docx]

**Supplemental Table 3. Sequencing Primers**

| **Name** | **Sequence** | **Location** |
| --- | --- | --- |
| ME0096 | CCAATGAGACTGGTGTCATTC | 5'UTR, forward |
| ME0097 | CAGCATAGCAAGAACCCATC | Intron 2, reverse |
| ME0098 | CCAAGAGGACCATTGAGGAAG | Exon 2, forward |
| ME0099 | GCTGTGGAGAAACAGTGTGAG | Intron 3, reverse |
| ME0100 | GGAAAGCCCACAAGAAGCTC | Exon 3, forward |
| ME0101 | AGCATGAGAATGCAGCTGTG | Intron 5, reverse |
| ME0103 | GAAGGAGCCTTTTGCTTGTC | Intron 7, reverse |
| ME0104 | CACTGAGACCACAGCAAACAC | Exon 7, forward |
| ME0105 | CCTCCACCACATTTTCACG | Intron 9, reverse |
| ME0106 | CACAGTCATCATTCCGAACC | Exon 9, forward |
| ME0107 | GACCAAGAAACTTTCGCTCC | Exon 10, reverse |
| ME0108 | TGTAAACACAGTGCTGCGAG | Exon 10, forward |
| ME0117 | CACCCTCTGCAGGAGAGC | Intron 6, forward |
| ME0118 | CTTGCTCAATGCCACCATC | Exon 8, forward |
| ME0152 | TAAAAACCTGGAGCCACTGG | Exon 5, forward |
| CAH-ME0001 | GGCCCGAATTTCTGAGTCAG | Exon 1, forward |
| CAH-ME0002 | CTTTGGGGCATCCCCAATCCAGG | Intron 2, forward |
